# ^11^C-PiB and ^124^I-Antibody PET Provide Differing Estimates of Brain Amyloid-β After Therapeutic Intervention

**DOI:** 10.2967/jnumed.121.262083

**Published:** 2022-02

**Authors:** Silvio R. Meier, Dag Sehlin, Sahar Roshanbin, Victoria Lim Falk, Takashi Saito, Takaomi C. Saido, Ulf Neumann, Johanna Rokka, Jonas Eriksson, Stina Syvänen

**Affiliations:** 1 Department of Public Health and Caring Sciences/Geriatrics, Uppsala University, Uppsala, Sweden;; 2 Laboratory for Proteolytic Neuroscience, RIKEN Center for Brain Science, Wako, Japan;; 3 Department of Neurocognitive Science, Institute of Brain Science, Nagoya City University Graduate School of Medical Sciences, Nagoya, Japan;; 4 Neuroscience Research, Novartis Institutes for BioMedical Research, Basel, Switzerland;; 5 Department of Medicinal Chemistry, Uppsala Biomedical Center, Uppsala University, Uppsala, Sweden; and; 6 PET Centre, Uppsala University Hospital, Uppsala, Sweden

**Keywords:** Alzheimer disease, BACE-1 inhibition, amyloid-β, ^11^C-PiB, antibody-based PET

## Abstract

PET imaging of amyloid-β (Aβ) has become an important component of Alzheimer disease diagnosis. ^11^C-Pittsburgh compound B (^11^C-PiB) and analogs bind to fibrillar Aβ. However, levels of nonfibrillar, soluble, aggregates of Aβ appear more dynamic during disease progression and more affected by Aβ-reducing treatments. The aim of this study was to compare an antibody-based PET ligand targeting nonfibrillar Aβ with ^11^C-PiB after β-secretase (BACE-1) inhibition in 2 Alzheimer disease mouse models at an advanced stage of Aβ pathology. **Methods:** Transgenic ArcSwe mice (16 mo old) were treated with the BACE-1 inhibitor NB-360 for 2 mo, whereas another group was kept as controls. A third group was analyzed at the age of 16 mo as a baseline. Mice were PET-scanned with ^11^C-PiB to measure Aβ plaque load followed by a scan with the bispecific radioligand ^124^I-RmAb158-scFv8D3 to investigate nonfibrillar aggregates of Aβ. The same study design was then applied to another mouse model, *App^NL-G-F^*. In this case, NB-360 treatment was initiated at the age of 8 mo and animals were scanned with ^11^C-PiB-PET and ^125^I-RmAb158-scFv8D3 SPECT. Brain tissue was isolated after scanning, and Aβ levels were assessed. **Results:** ^124^I-RmAb158-scFv8D3 concentrations measured with PET in hippocampus and thalamus of NB-360–treated ArcSwe mice were similar to those observed in baseline animals and significantly lower than concentrations observed in same-age untreated controls. Reduced ^125^I-RmAb158-scFv8D3 retention was also observed with SPECT in hippocampus, cortex, and cerebellum of NB-360–treated *App^NL-G-F^* mice. Radioligand in vivo concentrations corresponded to postmortem brain tissue analysis of soluble Aβ aggregates. For both models, mice treated with NB-360 did not display a reduced ^11^C-PiB signal compared with untreated controls, and further, both NB-360 and control mice tended, although not reaching significance, to show higher ^11^C-PiB signal than the baseline groups. **Conclusion:** This study demonstrated the ability of an antibody-based radioligand to detect changes in brain Aβ levels after anti-Aβ therapy in ArcSwe and *App^NL-G-F^* mice with pronounced Aβ pathology. In contrast, the decreased Aβ levels could not be quantified with ^11^C-PiB PET, suggesting that these ligands detect different pools of Aβ.

## Visual Abstract

**Figure:**
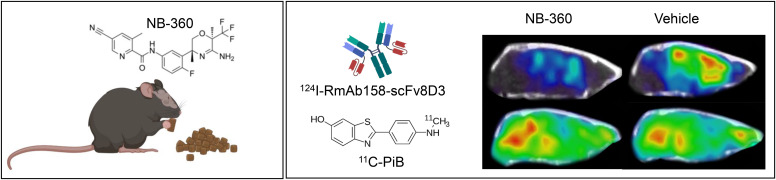

Alzheimer disease (AD) is a growing socioeconomic burden on society and health care in most countries that are characterized by an aging population (*1*). Despite intense research over the last few decades, no treatment is available that halts the underlying disease mechanisms and stops the pathologic changes in the AD brain. Accumulation of amyloid-β (Aβ) plaques is the core feature of the histopathologic diagnosis of AD and can be visualized and quantified by molecular imaging. PET is today a valuable tool for assessment of brain amyloidosis in vivo. Amyloid imaging with PET has also become a regularly used inclusion criterion for enrolment of patients in clinical trials. New treatments, aiming to clear Aβ from the brain parenchyma or to reduce Aβ production and aggregation, are dependent on diagnostic tools to follow changes in brain Aβ levels in vivo.

PET ligands such as ^11^C-Pittsburgh compound B (^11^C-PiB) and several later-developed analogs bind to fibrillar Aβ, that is, the form of Aβ found in insoluble amyloid plaques. However, Aβ aggregation starts years before any clinical symptoms emerge, and it appears that the PET signal with amyloid radioligands such as ^11^C-PiB becomes saturated rather early during disease progression (*2*,*3*). In contrast, nonfibrillar Aβ oligomers and protofibrils have been reported to display a more dynamic profile during the course of the clinical stages of the disease and may therefore be better biomarkers for disease severity than amyloid plaques (*3*,*4*). Treatments aimed at reducing brain Aβ, such as β-secretase (BACE-1) inhibitors, or to facilitate Aβ clearance, for example, immunotherapy, are likely to reduce nonfibrillar Aβ before amyloid plaques (*5*,*6*). Furthermore, diffuse Aβ plaque pathology cannot be detected by these radioligands, which bind to the ordered β-sheet structures of amyloid plaques (*7*).

A potential strategy to image nonfibrillar Aβ aggregates, rather than plaques, and thus a way to circumvent the limitations of ^11^C-PiB and other amyloid radioligands could be to use an antibody-based PET approach. Antibodies are characterized by high and specific binding to their target and can be generated to show selective affinity for a specific aggregation form of Aβ, for example, Aβ protofibrils (*8*). However, antibodies display very limited passage across the blood–brain barrier and are therefore not directly suitable as radioligands that require fast and efficient brain entry. We have recently introduced several bispecific radioligands based on Aβ-binding antibodies functionalized with a transferrin receptor binding component to enable active transport across the blood–brain barrier (*9*–*13*).

RmAb158-scFv8D3 (*14*) is based on the Aβ protofibril selective antibody mAb158 (*8*,*15*), the murine version of lecanemab (*16*) that is currently being evaluated as an anti-Aβ treatment in clinical phase III trials, and 2 single-chain fragments (scFvs) of the transferrin receptor antibody 8D3 (*17*) to enhance brain uptake. A previous study showed that PET with ^124^I labeled RmAb158-scFv8D3 could be used to successfully follow Aβ accumulation in mice 7–16 mo of age harboring the Arctic (Aβ precursor protein [APP] E693G) and the Swedish (APP KM670/671NL) APP mutations (ArcSwe) (*18*). Further, ^124^I-RmAb158-scFv8D3 also enabled monitoring of Aβ brain levels after Aβ-reducing treatment with BACE-1 inhibitor NB-360 (*6*,*19*) in a cross-sectional study design in ArcSwe mice 10 mo old, that is, an age associated with limited Aβ accumulation. However, in the clinical situation, it is likely that most AD cases remain undetected until clinical symptoms such as memory impairment appear. Consequently, a disease-modifying treatment will realistically be applied at a disease stage associated with advanced brain Aβ accumulation. Thus, diagnostic and dynamic biomarkers reflecting pathologic changes covering also the middle to late disease stage are required.

The aim of this study was to compare the ability of the clinically established radioligand ^11^C-PiB and the novel protofibril selective radioligand ^124^I-RmAb158-scFv158 to detect and quantify effects of anti-Aβ intervention using the BACE-1 inhibitor NB-360 as a model drug. The study was performed on 2 different models: the first was the ArcSwe mouse model that shows ^11^C-PiB positivity between the ages of 12 and 18 mo (*9*,*20*), and the second was the *App^NL-G-F^* knock-in mouse model harboring the Arctic, Swedish, and Iberian (APP I716F) mutations that is characterized by diffuse Aβ pathology that is not readily detected by amyloid imaging with PET (*21*). By inclusion of old mice characterized by abundant brain Aβ pathology, the study was designed to resemble the disease stage when patients are likely to be diagnosed and potentially enrolled into clinical trials of novel drug candidates.

## MATERIALS AND METHODS

### Animals and Treatment

All experiments were performed according to the rules and regulations of the Swedish Animal Welfare Agency, which have been in line with the European Communities Council Directive since September 22, 2010. The experiments were approved by the Uppsala University Animal Ethics board (5.8.18-13350/2017). ArcSwe mice (*22*) 16 mo old were administered BACE-1 inhibitor NB-360 (Novartis) (*6*) nutrition pellets (0.5 g of NB-360/kg of pellets) for 2 mo. *App^NL-G-F^* mice (*23*), with an earlier onset of Aβ deposition, were treated between the ages of 8 and 10 mo. NB-360–treated groups were compared with age-matched groups that received only vehicle food, and further, with baseline groups reflecting pathology levels at the beginning of the treatment. In total, 44 ArcSwe mice (baseline, *n* = 15; NB-360, *n* = 15; control, *n* = 14) and 17 *App^NL-G-F^* mice (baseline, *n* = 5; NB-360, *n* = 6; control, *n* = 6) were included in the study. Two wild-type mice 8 mo old, that is, age-matched to the *App^NL-G-F^* baseline mice, were also included as a comparison (study design is shown in Supplemental Fig. 1 and animal information in Supplemental Table 1; supplemental materials are available at http://jnm.snmjournals.org). In addition to the mice that underwent in vivo imaging, a separate group of mice, ArcSwe (*n* = 2; 18 mo old) and *App^NL-G-F^* (*n* = 2; 10 mo old) were used for ex vivo autoradiography. Mice had free access to food and water during the study.

### Radiochemistry

^11^C-PiB was synthesized using a previously described method with slight modifications related to automation using an in-house–built synthesis device (Tracer Production System) (*24*). The final product was reformulated using solid-phase extraction in approximately 10% ethanol in phosphate-buffered saline. ^11^C-PiB was produced with a radioactivity yield of 2.1 ± 1.0 GBq (range, 0.7–4.3 GBq), a molar activity of 33 ± 38 MBq/nmol, and a radiochemical purity of more than 99% at the end of the synthesis.

### Antibody Labeling

RmAb158-scFv8D3 was labeled using direct radioiodination (*25*) as previously described (*18*). ^124^I (PerkinElmer Inc.) labeling was done in 8 batches; 80 μg of RmAb158-scFv8D3 were labeled with 101.9 ± 16.6 MBq, resulting in an average yield of about 75.7% ± 2.5%. A similar procedure was used for ^125^I labeling of RmAb158-scFv8D3 (*26*); 80 μg of RmAb158-scFv8D3 were labeled with 38.2 ± 4.3 MBq of ^125^I, resulting in an average yield of 71.7% ± 3.6%.

### PET/SPECT Imaging

All mice underwent an ^11^C-PiB PET scan. ArcSwe mice were injected with 13.2 ± 3.6 MBq of ^11^C-PiB with a molar activity of 19.0 ± 9.3 MBq/nmol. *App^NL-G-F^* mice were injected with a 20.1 ± 6.6 MBq/nmol concentration of ^11^C-PiB with a molar activity of 6.7 ± 1.6 MBq/nmol. Animals were either injected at the start of the PET scan and scanned for 1 h or injected 30 min before the PET scan and kept under anesthesia until the start of a 30-min scan. For all animals, ^11^C-PiB brain retention was analyzed using data acquired 40–60 min after injection.

Within a week after their ^11^C-PiB PET scan, ArcSwe animals were PET-scanned with ^124^I-RmAb158-scFv8D3 and *App^NL-G-F^* mice were SPECT-scanned with ^125^I-RmAb158-scFv8D3. One day before injection with radiolabeled RmAb158-scFv8D3, mice were given drinking water containing 0.5% NaI to reduce thyroidal uptake of ^124^I and ^125^I. After injection, the concentration was decreased to 0.2% NaI until the PET or SPECT scan. ArcSwe and *App^NL-G-F^* mice were injected with 11.6 ± 2.7 MBq of ^124^I-RmAb158-scFv8D3 and 7.2 ± 1.1 MBq of ^125^I-RmAb158-scFv8D3, respectively, and scanned 4 d after injection. The molar activities were 185.4 ± 28.7 MBq/nmol and 144.5 ± 8.8 MBq/nmol for the ^124^I- and the ^125^I-labeled radioligands, respectively. After PET/SPECT scanning, mice underwent transcardial perfusion with 40 mL of 0.9% NaCl for 2.5 min. The brain was then isolated and divided into right and left hemispheres, and the cerebellum was removed from the left hemisphere. Radioactivity was measured in the 3 brain samples (right hemisphere, left hemisphere without cerebellum, and cerebellum from the left hemisphere) with a Wizard 2470 γ-counter (GE Healthcare). All samples were frozen on dry ice and stored at −80°C until further processing.

PET scans were performed on either a Triumph Trimodality System (TriFoil Imaging, Inc.) or a nanoScan system PET/MRI (Mediso). All PET scans performed with the Mediso system were reconstructed with a Tera-Tomo 3-dimensional algorithm (Mediso) with 4 iterations and 6 subsets. Data obtained with the Triumph system were reconstructed using 3-dimensional ordered-subsets expectation maximization with 20 iterations. SPECT scans were performed with a nanoScan SPECT/CT system (Mediso) with 4 detectors at a frame time of 80 s. Images were reconstructed with a Tera-Tomo 3-dimensional algorithm (Mediso) with 48 iterations and 3 subsets. Each mouse was CT-examined after the PET/SPECT scan.

All subsequent processing of the images was performed with Amide, version 1.0.4 (*27*). CT and PET scans were manually aligned with a T2-weighted mouse brain atlas (*28*) to quantify activity in regions of interest (Supplemental Fig. 2).

### Immunostaining and Autoradiography

Right brain hemispheres of PET- or SPECT-scanned animals were cryosectioned (20 μm) for anti-Aβ1-42 chromogen staining as described previously (*18*) using the primary polyclonal rabbit-anti-Aβ1-42 antibody (Agrisera). Triple immunofluorescence staining of Aβ, ionized calcium binding adaptor molecule 1, and glial fibrillary acidic protein and autoradiography were performed as previously described (*18*). Images were processed as described by Gustavsson et al. (*26*).

### Brain Sample Preparation

Brain tissue was sequentially extracted as previously described (*29*) according to Table 1, using a Precellys Evolution system (Bertin Corp.) (4 × 10 s at 5,500 rpm).

**TABLE 1:** Extractions Performed on Brain Tissue for ELISA Analysis

Step	Material	Extraction	Medium	Centrifugation
1	Fresh-frozen brain tissue	1:5 weight:volume ratio of tissue	TBS	1 h, 16,000*g*
2	Pellet TBS extraction (step 1)	1:5 weight:volume ratio of tissue	70% formic acid	1 h, 16,000*g*
3	TBS extract (step 1)	200 μL of TBS extract (step 1)	TBS	1 h, 100,000*g*

### Biochemical Quantifications of Brain Tissue

Brain extraction samples (Table 1) were quantified with enzyme-linked immunosorbent assay (ELISA) as previously described (*20*,*30*). Assay details are displayed in Table 2.

**TABLE 2:** Antibodies and Extraction Fractions Used in ELISA Analysis

Target	Extraction sample	Primary antibody	Secondary antibody	Distributor
Nonfibrillar Aβ aggregates	TBS, 16,000*g*	mAb3D6	mAb3D6-bio	In-house expression
Small sized, nonfibrillar Aβ aggregates	TBS, 100,000*g*	mAb3D6	mAb3D6-bio	In-house expression
Fibrillar Aβ1-40	FA, 16,000*g*	Anti-Aβ40	mAb3D6-bio	Agrisera/in-house expression
Fibrillar Aβ1-42	FA, 16,000*g*	Anti-Aβ42	mAb3D6-bio	Invitrogen/in-house expression
sTREM2	TBS, 16,000*g*	AF1729	BAF1729	R&D Systems

FA = formic acid.

### ^11^C-PiB Nuclear Track Emulsion (NTE) and Autoradiography

A separate group of mice was injected with 18–20 MBq of ^11^C-PiB and then underwent transcardial perfusion at 20 or 40 min after injection. The brain was immediately removed and divided into right and left hemispheres. Brain samples were frozen on dry ice and processed into 20-μm sagittal sections for NTE and 40-μm sections for ex vivo autoradiography. Before NTE, sections were stained for 2 min with saturated thioflavin-S in 80% ethanol, washed 1 min in 70% ethanol, and rinsed with phosphate-buffered saline. NTE was performed as previously described (*29*). Exposure of the slides was started 30 min after perfusion (i.e., equal to 1.5 decay half-lives of ^11^C). The signal was developed after 2 h. Images were acquired with an LSM700 confocal laser scanning microscope (Zeiss) and processed with Zen Zeiss software. Images were compiled with Adobe Photoshop 2020. Brain sections from the same animals were also exposed to a phosphor imaging plate (Fujifilm) within 20 min after perfusion. Plates were exposed for 80 min and read with an Amersham Typhoon imager (GE Healthcare).

### Statistics

Data were analyzed and plotted with GraphPad Prism, version 6. Groups were compared with 1-way ANOVA using the Bonferroni post hoc test. Results are reported as mean ± SD.

## RESULTS

**FIGURE 1.**
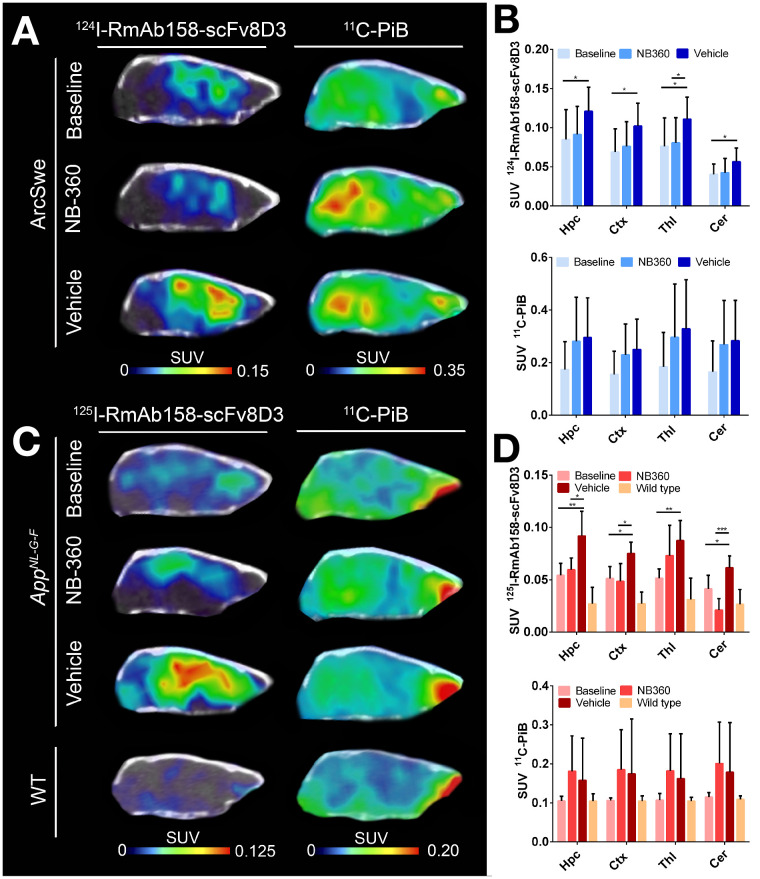
PET images and quantification of ^11^C-PiB scans (40–60 min after injection) and ^124^I-RmAb158-scFv8D3 scans (72 h after injection) expressed as SUV. (A) Comparison of representative ^124^I-RmAb158-scFv8D3 and ^11^C-PiB PET images in ArcSwe animals. (B) Quantification of ^124^I-RmAb158-scFv8D3 and ^11^C-PiB in hippocampus (Hpc), cortex (Ctx), thalamus (Thl), and cerebellum (Cer). (C) Comparison of representative SPECT and PET images of ^125^I-RmAb158-scFv8D3 and ^11^C-PiB in *App^NL-G-F^* and wild-type animals. (D) Retention of ^125^I-RmAb158-scFv8D3 and ^11^C-PiB in different brain regions of *App^NL-G-F^* and wild-type animals.

ArcSwe and *App^NL-G-F^* mice, treated with BACE-1 inhibitor NB-360 or with vehicle, were PET-scanned with ^11^C-PiB followed by a ^124^I-RmAb158-scFv8D3 PET scan or a ^125^I-RmAb158-scFv8D3 SPECT scan. On the basis of visual interpretation of PET images, ^11^C-PiB retention in ArcSwe animals seemed slightly increased in the NB-360 and vehicle groups compared with the 2-mo-younger baseline group (Fig. 1A). When retention was quantified as SUV, a similar trend was observed in hippocampus, cortex, thalamus, and cerebellum, but the difference was not significant and interanimal variation was large (Fig. 1B). ^11^C-PiB retention in *App^NL-G-F^* mice was alike in all 3 groups (Fig. 1C). When retention was quantified as SUV, interindividual variation was high and differences between the 3 groups and the wild-type group were not significant (Fig. 1D). In summary, neither of the mouse models showed a significant difference in ^11^C-PiB signal between the different groups, despite a trend toward an increased signal in older mice, that is, after the 2-mo treatment period (both vehicle and NB-360), compared with baseline mice. Whole-body PET images are shown in Supplemental Figure 3.

^124^I-RmAb158-scFv8D3 retention in NB-360–treated animals was clearly lower than in vehicle animals, whereas there was no notable difference from baseline animals (Fig. 1A). Radioligand concentrations were significantly lower in the thalamus (*P* = 0.049) of NB-360–treated animals than in vehicle animals (Fig. 1B). The same trend was observed in cortex, hippocampus, and cerebellum but did not reach significance. Vehicle animals displayed increased levels compared with baseline (hippocampus, *P* = 0.028; cortex, *P* = 0.018; thalamus, *P* = 0.021; cerebellum, *P* = 0.039). Akin to results in ArcSwe animals, SPECT images revealed lower ^125^I-RmAb158-scFv8D3 retention in *App^NL-G-F^* animals treated with NB-360 than in the vehicle group (Fig. 1C). When quantified, radioligand concentration was significantly lower in hippocampus (*P* = 0.017), cortex (*P* = 0.047), and cerebellum (*P* < 0.001) (Fig. 1D). Vehicle animals displayed increased ^125^I-RmAb158-scFv8D3 concentrations in hippocampus (*P* = 0.008) and thalamus (*P* = 0.047) compared with baseline.

**FIGURE 2.**
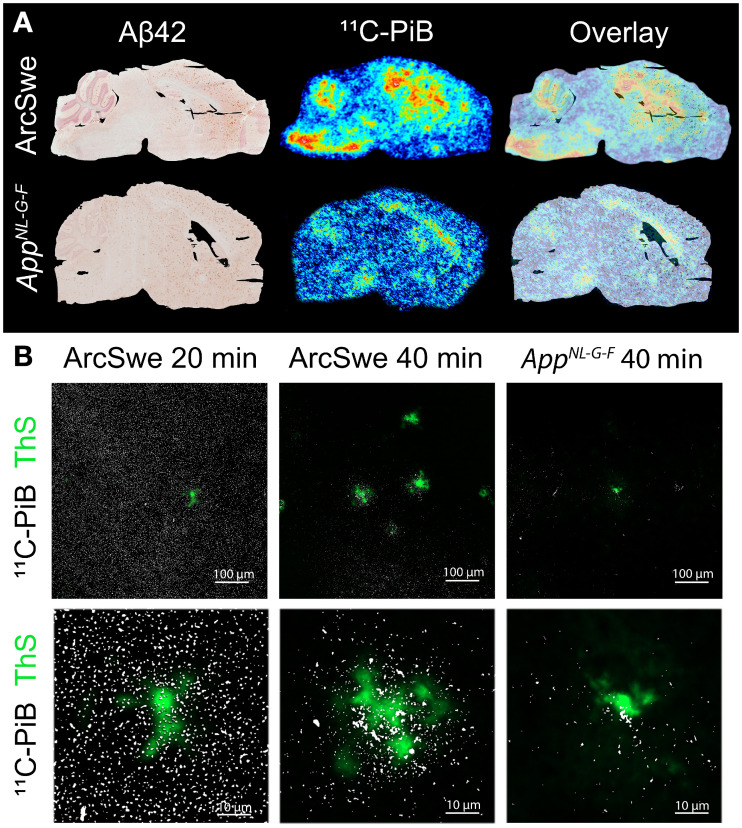
Ex vivo ^11^C-PiB retention in postmortem brain tissue. (A) Aβ42-stained brain sections of 18-mo-old ArcSwe mouse and 10-mo-old *App^NL-G-F^* mouse and corresponding ex vivo ^11^C-PiB autoradiography images at 40 min after radioligand injection. Overlay illustrates overlap of regions with abundant Aβ plaque pathology and radioligand binding. (B) Thioflavin S staining (green) and NTE (white dots) of ^11^C-PiB in ArcSwe and *App^NL-G-F^* mice.

^11^C-PiB binding was also assessed in postmortem brain tissue with ex vivo autoradiography and compared with Aβ42 immunostaining of the adjacent brain sections (Fig. 2A). At 40 min after injection, ArcSwe animals showed ^11^C-PiB binding in regions with abundant Aβ pathology such as hippocampus, cortex, and thalamus. White matter binding was observed in cerebellum, corpus callosum, pons, and medulla (Fig. 2A). *App^NL-G-F^* mice displayed low ^11^C-PiB binding in hippocampus, cortex, and thalamus despite Aβ pathology but. in line with observations in ArcSwe animals, also showed distinct white matter binding. ^11^C-PiB binding in the cortex was further investigated with NTE (Fig. 2B). At 20 min after ^11^C-PiB injection in ArcSwe mice, the radioligand was evenly distributed in the tissue, including the core of thioflavin S–stained Aβ deposits, whereas at 40 min after injection, the radioligand was localized primarily around the dense core of thioflavin S–stained Aβ plaques. ^11^C-PiB retention in *App^NL-G-F^* mice at 40 min after injection was lower than that observed in ArcSwe brain but, when present, also localized around the cores of thioflavin S–positive Aβ deposits.

**FIGURE 3.**
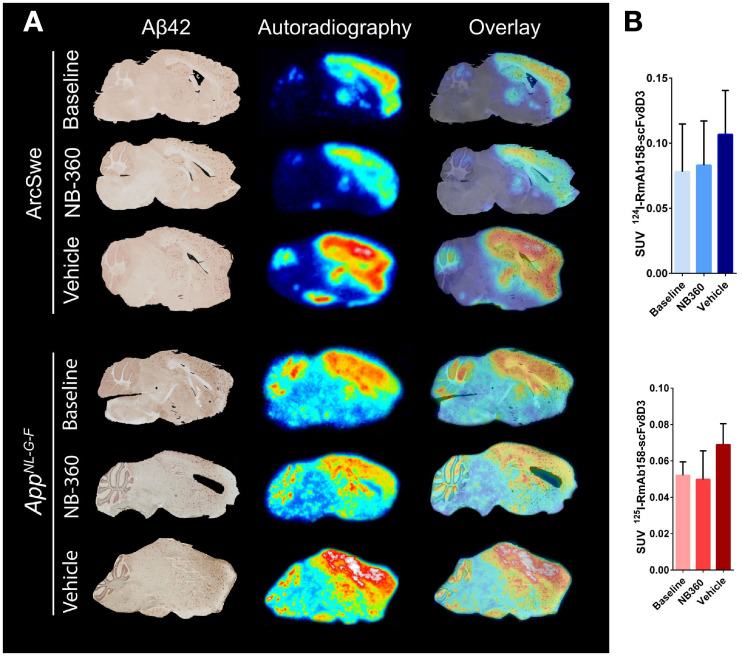
Aβ42 immunohistochemistry and ex vivo autoradiography of ^124/125^I-RmAb158-scFv8D3 in brain tissue. (A) Comparison of Aβ42 staining and autoradiography on sagittal brain sections of 1 representative ArcSwe or *App^NL-G-F^* animal of each studied group. Stained brain section was merged to overlay with corresponding ex vivo autoradiography of same animal to visualize pathology and tracer binding simultaneously. (B) Postmortem ex vivo quantification of ^124/125^I-RmAb158-scFv8D3 in complete right hemisphere in ArcSwe and *App^NL-G-F^* animals.

Ex vivo autoradiography with radiolabeled RmAb158-scFv8D3 visualized the presence of the ligand in most parts of the brain. There was especially high retention of the radioligand in cortex, hippocampus, and thalamus already in the baseline groups in both ArcSwe and *App^NL-G-F^* mice (Fig. 3A). The spatial distribution of the radioligand did not change because of NB-360 or vehicle treatment, but the intensity of the radioactive signal was lower in the NB-360 and baseline ArcSwe and *App^NL-G-F^* mice than in vehicle-treated animals. This trend was also evident when the complete postmortem right hemispheres (from which brain sections were prepared) were measured in a γ-counter, although the difference did not reach significance because of large interindividual variation (Fig. 3B). Aβ42 staining visualized Aβ-affected brain regions, and further, the overlap between pathology-rich brain regions and radiolabeled RmAb158-scFv8D3 strongly indicated a colocalization between the radioligand and Aβ-affected regions in both mouse models (Fig. 3A). NTE in combination with triple staining of glial fibrillary acidic protein, ionized calcium binding adaptor molecule 1, and Aβ is shown in the Supplemental Figure 4.

**FIGURE 4.**
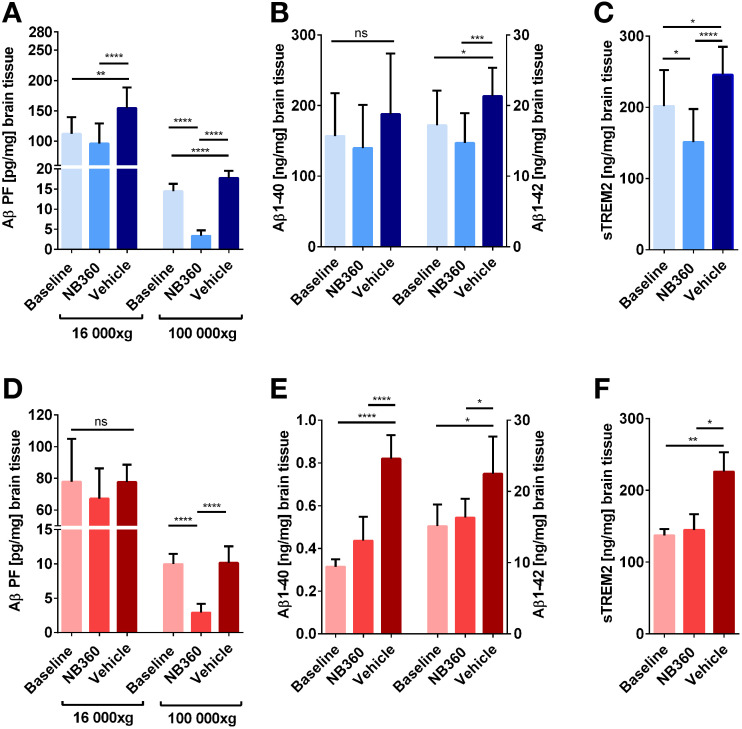
Quantification of Aβ and sTREM2 in brain homogenates. (A) Quantification of nonfibrillar, TBS-soluble Aβ aggregates separated at 16,000g and 100,000g from ArcSwe brain homogenates. (B) Insoluble Aβ1-40 and Aβ1-42 in the formic acid fraction of ArcSwe brain homogenates. (C) sTREM2 levels in the TBS fraction of ArcSwe brain homogenates. (D) Quantification of nonfibrillar, TBS-soluble Aβ aggregates separated at 16,000g and 100,000g from *App*^*NL-G-F*^ brain homogenates. (E) Insoluble Aβ1-40 and Aβ1-42 in the formic acid fraction of *App*^*NL-G-F*^ brain homogenates. (F) sTREM2 levels in the TBS fraction of *App*^*NL-G-F*^ brain homogenates.

Brain homogenates of all animals that underwent PET or SPECT were biochemically assessed with ELISA. Tris-buffered saline (TBS)–soluble Aβ aggregates were quantified after centrifugation at 16,000*g* and 100,000*g* (Figs. 4A and 4D). In the 16,000*g* fractions, NB-360–treated ArcSwe animals showed lower levels of Aβ aggregates than did the vehicle group (*P* = 0.0029), whereas this difference was not significant in the *App^NL-G-F^* mice (*P* > 0.99). However, this decrease was more distinctive and significant in both animal models in the 100,000*g* fraction (*P* < 0.0001) representing smaller and more soluble aggregates. In addition, the NB-360 groups displayed lower Aβ levels in 100,000*g* fractions than did the baseline groups (*P* < 0.0001). Aβ1-40 and Aβ1-42 in the formic acid fraction represent TBS-insoluble Aβ, including fibrils, and thus represent total plaque load (Figs. 4B and 4E). NB-360–treated *App^NL-G-F^*, but not ArcSwe, displayed lower Aβ1-40 levels than vehicle-treated animals, whereas Aβ1-42 levels were decreased in NB-360–treated animals compared with vehicle animals in both models. Correlations between PET/SPECT SUV and Aβ levels are included in Supplemental Tables 2–5. Microglial activation was assessed by quantification of soluble triggering receptor expressed on myeloid cells 2 (sTREM2) in the 16,000*g* fraction (Figs. 4C and 4F). BACE-1 inhibition decreased sTREM2 levels compared with vehicle in both models (*P* < 0.0001). In the ArcSwe animals, which showed higher sTREM2 levels than the *App^NL-G-F^* animals at baseline, NB-360 treatment also reduced sTREM2 levels compared with baseline (*P* = 0.0143).

## DISCUSSION

Amyloid imaging has become an important inclusion criterion in clinical trials of candidate drugs aimed at reducing brain Aβ. Established amyloid radioligands, such as ^11^C-PiB, bind to Aβ fibrils deposited as insoluble plaques in the AD brain. These established radioligands may therefore be insufficient for monitoring changes in more soluble or diffuse forms of misfolded and aggregated Aβ, which are likely to be affected first by anti-Aβ drugs. In this study, we demonstrated that radiolabeled bispecific antibody RmAb158-scFv8D3, binding to soluble Aβ aggregates, was able to quantify changes in brain Aβ levels after treatment with BACE-1 inhibitor NB-360 in 2 mouse models of Aβ pathology and, further, that the readout was different from that of ^11^C-PiB PET, which did not detect any differences between treated and untreated groups.

The NB-360 treatment was started at an age when Aβ brain pathology was already advanced and the brain tissue, at least in the ArcSwe mice, included large amounts of dense-core Aβ deposits. Thus, it may not be surprising that the ^11^C-PiB signal did not decrease with treatment, as these deposits are likely to be difficult to dissolve. In line with this observation, formic acid–soluble Aβ1-40, the main constituent of dense-core deposits (*31*), displayed the smallest difference between treatment groups. However, it was somewhat surprising that despite BACE-1 inhibition, leading to a dramatic reduction of the smallest aggregates as shown by ELISA in the 100,000*g* TBS fraction, the ^11^C-PiB signal tended to increase from baseline to the end of treatment. This findings implies that once insoluble deposits have been formed, they may continue to increase in number and size, especially if the pool of monomers and nonfibrillar aggregates has not been completely depleted. As illustrated by the ELISA measurements, the reduction in intermediate-sized Aβ aggregates, that is, the 16,000*g* fraction, was either smaller than that observed for the soluble aggregates in the 100,000*g* fraction (ArcSwe) or absent (*App^NL-G-F^*). A longer treatment time may be required to remove also the 16,000*g* aggregates. This hypothesis is supported by clinical studies of BACE-1 inhibitors that have reported decreased brain amyloid levels detected with PET after 1.5–2 y of treatment (*32*,*33*).

The spatial distribution of ^124^I-RmAb158-scFv8D3 studied by ex vivo autoradiography in combination with Aβ42 immunohistochemistry indicated radioligand accumulation in Aβ-rich brain regions in both mouse models. In contrast, ex vivo autoradiography with ^11^C-PiB was evident in regions with abundant Aβ pathology only in the ArcSwe model, not in the *App^NL-G-F^* model. The main reason for selecting these 2 models for the present study was their dissimilar Aβ profiles, illustrated by their very different relative ratios of Aβ40 and Aβ42; Aβ40 is the major Aβ species in ArcSwe mice, whereas Aβ42 dominates in *App^NL-G-F^* mice (Fig. 4). It has been shown that although Aβ42 is more prone to aggregate, the dense core of plaques is formed by Aβ40 (*31*). It should also be noted that Aβ40 is the major Aβ isoform produced in human sporadic AD (*34*). Thus, this fact leads to another important aspect highlighted in the present study, that is, the selection of animal models for preclinical studies of brain Aβ, especially when evaluating the ability of candidate drugs to reduce pathologic changes. The application of ^11^C-PiB, and analogs, in animal studies has indeed been debated over the last 10–15 y. First, preclinical attempts to quantify Aβ deposits with ^11^C-PiB in the PS1/APP transgenic mouse model resulted in contradictory results claiming structural differences between Aβ plaque formation and cerebral pathology in mice and humans (*35*). Yet, more recent studies have demonstrated that Aβ deposits can be assessed by ^11^C-PiB in mouse models such as APP23 (*36*,*37*) and APP/PS1-21 (*38*). Further, several studies with ^18^F-labeled analogs of ^11^C-PiB have underlined the ability of Aβ plaque assessment in different mouse models (*39*), especially in longitudinal studies (*21*,*40*). Several studies have reported the ability of amyloid PET to quantify disease-modifying treatments, for example, mApoE-pA-Lip in APP23 mice (*41*) and BACE-1 inhibition in PS2APP mice (*42*). Thus, the use of amyloid PET likely requires a model with dense-core Aβ deposits. The present study also demonstrated that weak ^11^C-PiB binding is not per se a sign of low brain Aβ levels, as radiolabeled RmAb158-scFv8D3 was readily able to detect the abundant Aβ pathology in ^11^C-PiB–negative *App^NL-G-F^* mice both in vivo and ex vivo. In line with this observation, patients with AD caused by specific mutations in the AβPP, with confirmed diffuse pathology and absence of dense-core plaques, have also been reported as ^11^C-PiB–negative (*7*). Again, this finding illustrates the need for radioligands able to quantify Aβ in forms other than insoluble deposits (plaques).

We used SUV, that is, activity concentrations normalized to the injected activity per body weight, as the main readout measure from PET. This is different from most studies that have reported SUVRs—that is, activity ratios between regions of interest and a reference region. The reference region used in previous studies has in most cases been cerebellum or periaqueductal gray matter (*21*). However, in the present study, Aβ pathology was spread in the whole brain at the start of the study, hence excluding the use of a pathology-free region as a reference. In addition, all brain regions, including cerebellum and periaqueductal gray matter, were affected by disease progression and by NB-360 treatment as shown by PET/SPECT and autoradiography and by ELISA of postmortem cerebellum homogenates (Supplemental Fig. 5). Thus, in this setting it was not possible to use reference region–based methods.

Apart from Aβ, brain sTREM2 concentrations were also investigated in brain homogenates and found to be decreased in both mouse models after administration of NB-360. This finding suggests an extenuating effect on microglia activation due to lower Aβ production and aggregation.

## CONCLUSION

Antibody-based PET and SPECT imaging of soluble Aβ aggregates is a sensitive tool to follow Aβ pathology in the brain. This study demonstrated the ability of such ligands to quantify changes due to anti-Aβ treatment at a stage of advanced Aβ pathology. Thus, radioligands based on antibodies directed toward a specific form of aggregated Aβ may have potential to improve and complement diagnostics in preclinical and clinical studies of AD drug candidates. We demonstrated in this study that radiolabeled RmAb158-scFv8D3 is able to quantify changes in brain Aβ levels after BACE-1 inhibition in 2 AD mouse models, and further, that the readout is different from that of ^11^C-PiB.

## DISCLOSURE

This work was supported by grants from the Swedish Research Council (2017-02413 and 2018-02715), Alzheimerfonden, Hjärnfonden, Torsten Söderbergs stiftelse, Åhlénstiftelsen, Magnus Bergwalls stiftelse, Stiftelsen för gamla tjänarinnor, and Konung Gustaf V:s och Drottning Victorias Frimurarestiftelsen. The funding bodies did not take part in design of the study; in collection, analysis, or interpretation of data; or in writing of the manuscript. The molecular imaging work in this study was performed at the SciLifeLab Pilot Facility for Preclinical PET-MRI, a Swedish nationally available imaging platform at Uppsala University, Sweden, financed by the Knut and Alice Wallenberg Foundation. Ulf Neumann is an employee and shareholder of Novartis Pharma AG, Basel, Switzerland. No other potential conflict of interest relevant to this article was reported.

## Notes

KEY POINTS **QUESTION:** Do ^11^C-PIB and ^124^I-antibody PET readouts provide differing estimates of brain Aβ after therapeutic intervention in ArcSwe and *App^NL-G-F^* mice with pronounced Aβ pathology, and if they do, what is the implication for drug development for AD? **PERTINENT FINDINGS:** The antibody-based radioligand detected changes in brain Aβ levels after anti-Aβ therapy in ArcSwe and *App^NL-G-F^* mice. In contrast, the decreased Aβ levels could not be quantified with gold-standard ^11^C-PiB PET, suggesting that these ligands detect different pools of Aβ. **IMPLICATIONS FOR PATIENT CARE:** Radioligands based on antibodies directed toward a specific form of aggregated Aβ may have potential to improve and complement diagnostics in preclinical and clinical studies of AD drug candidates.
